# Search for Partner Proteins of *A. thaliana* Immunophilins Involved in the Control of Plant Immunity

**DOI:** 10.3390/molecules23040953

**Published:** 2018-04-19

**Authors:** Inna A. Abdeeva, Gennady V. Pogorelko, Liliya G. Maloshenok, Maria V. Mokrykova, Oksana V. Fursova, Sergey A. Bruskin

**Affiliations:** 1 N.I. Vavilov Institute of General Genetics Russian Academy of Sciences, Gubkina Str. 3, Moscow 119333, Russian; maloshenoklg@mail.ru (L.G.M.); marja-2007@yandex.ru (M.V.M.); brouskin@vigg.ru (S.A.B.); 2 Department of Plant Pathology and Microbiology, Iowa State University, Ames, IA 50011, USA; 3 Faculty of Geology, Lomonosov Moscow State University, GSP-1, 1 Leninskiye Gory, Moscow 119991, Russian; oksfursova@yandex.ru

**Keywords:** plant immunophilins, *A. thaliana*, plant immunite, yeast 2-hybrid, molecular partners of PPIases

## Abstract

The involvement of plant immunophilins in multiple essential processes such as development, various ways of adapting to biotic and abiotic stresses, and photosynthesis has already been established. Previously, research has demonstrated the involvement of three immunophilin genes (*AtCYP19-1/ROC3*, *AtFKBP65/ROF2*, and *AtCYP57*) in the control of plant response to invasion by various pathogens. Current research attempts to identify host target proteins for each of the selected immunophilins. As a result, candidate interactors have been determined and confirmed using a yeast 2-hybrid (Y2H) system for protein–protein interaction assays. The generation of mutant isoforms of ROC3 and AtCYP57 harboring substituted amino acids in the in silico-predicted active sites became essential to achieving significant binding to its target partners. This data shows that ROF2 targets calcium-dependent lipid-binding domain-containing protein (At1g70790; AT1) and putative protein phosphatase (At2g30020; АТ2), whereas ROC3 interacts with GTP-binding protein (At1g30580; ENGD-1) and RmlC-like cupin (At5g39120). The immunophilin AtCYP57 binds to putative pyruvate decarboxylase-1 (Pdc1) and clathrin adaptor complex-related protein (At5g05010). Identified interactors confirm our previous findings that immunophilins *ROC3*, *ROF2*, and *AtCYP57* are directly involved with stress response control. Further, these findings extend our understanding of the molecular functional pathways of these immunophilins.

## 1. Introduction

In the course of evolution, plants have developed various ways to adapt to and counter many biotic and abiotic stresses using physiological and molecular mechanisms. The main molecular tools utilized by plants to resist stress are the activation of specific ion channels; accumulation of reactive oxygen species (ROS) [1,2]; activation of protective kinase cascades [3]; and changes in the synthesis of phytohormones such as abscisic acid (ABA), salicylic acid (SA), jasmonic acid (JA), and ethylene (ET) [4]. Subsequent signal transduction alters the plant’s metabolism to activate defense mechanisms, regardless of the nature of the stress [5,6].

Quite interesting is the timing of the protective response process in the plant cell. The first reaction, immediately after the invasion of the pathogen or introduction of abiotic stress, is an alteration in the ion exchange fluxes across the cell membrane, primarily an increase in the influx of H^+^ and Ca^2+^ ions and outflow of K^+^ and anions [7,8]. Almost all abiotic stresses in plant cells temporarily increase the concentration of free cytosolic Ca^2+^ in the first seconds of the response [9,10]. A little later, approximately two minutes after the initial perception of the stress-related signal by the cell, an oxidative burst begins to develop. ROS have long been known to include compounds with strong oxidative properties that destroy the cell. However, it has been also shown that if large concentrations of ROS lead to cell death, lower amounts regulate the cellular response processes under stressful conditions [11,12,13]. This is one of the main functions of ROS: to serve as signaling molecules in cells [14,15].

The next stage of the response begins, on average, about 10 min after the receptors of the cell recognize the presence of a pathogen. It is then that the induction of MAP kinase cascades begins, accompanied by significant changes in the phosphorylation of host proteins. This leads to changes in their activity and ability to form complexes [16]. The culmination of this early response is associated with the activation of WRKY-type MAP kinase transcription factors through cascades, which leads to a change in cell metabolism and primary protective processes. Approximately 15 min later, the fastest transcriptional changes regulated by phytohormones appear, and as a result the expression of thousands of genes becomes altered. The main stress phytohormone is SA, which has been shown to be most active an average of one hour after reception of the initial stress signal. In addition, the earliest changes in the molecular-genetic apparatus of a plant cell in response to stress of any nature are also associated with post-translational modifications of proteins.

The study focuses on peptidyl-prolyl cis/trans isomerases (PPIase), a group of enzymes providing cis/trans isomerization of the polypeptide bond preceding the proline residue. So far, the functions of plant immunophilins and their role in pathogenesis have been poorly studied. In general, plant immunophilins are involved in basic immunity control, provided by the ability to catalyze isomerization in the polypeptide chains around proline residues via the action of enzymes with peptidyl-prolyl cis/trans isomerase (PPIase) activity [17,18].

Previously, three *A. thaliana* immunophilins were analyzed: *ROC3*, *ROF2*, and *AtCYP57* [1], whose expression level increased dramatically during the pathogenesis of the plant. This gave us the opportunity to assume their essential role in the immunity of plants. After determination of the localization within the plant cell of these PPIases and establishing their participation in the immune response against the bacterial phytopathogen *P. siryngae*, the greatest interest was to identify their interaction partners within the plant proteome, and as a consequence, shed light on the molecular pathway functions of these PPIases.

## 2. Results

### 2.1. ROF2 Interaction Partners

For the ROF2 immunophilin (encoded by *At5g48570*), 175 clones were obtained during the Y2H screening. Of the 175 clones, 42 were able to grow on Quadruple dropout medium (QDO) medium after replating. The list of clones containing the significant region of plant cDNA within the matching ORF is presented in Table 1.

**Figure 1:**
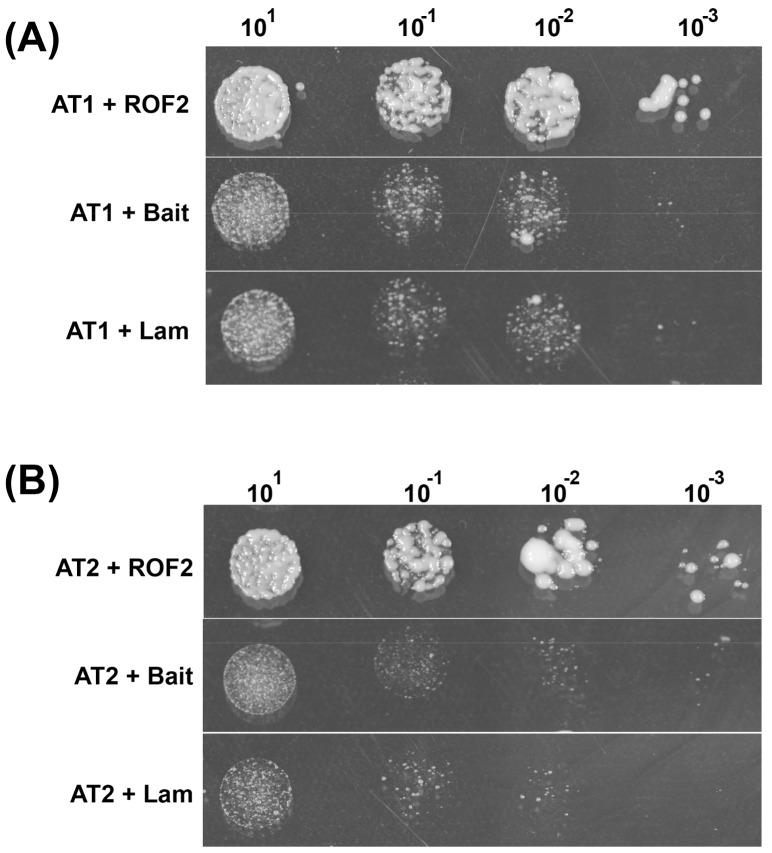
Cotransformation of ROF2 with AT1 (**A**) and AT2 (**B**) yeast cells (AH109 strain), and two negative controls: the empty “bait” of empty pGBKT7 vector and pGBKT7 carrying the control gene of the human protein lamin C (Lam). The initial culture of OD600 = 0.2 was used, sequentially diluted to 10^−3^.

Thus, in the first stage of Y2H, we obtained three potential candidate proteins for interaction with ROF2. Proteins that are part of the ribosome and the histone protein H2A_12 were shown to bind to other proteins analyzed previously (data not shown) and were considered to be the false positive interactors, so for further analysis we selected three other candidates: AT1, AT2, and AT4. Their physical interaction with ROF2 was confirmed by cotransformation. Figure 1А,B shows the results of interaction between ROF2 and the two target proteins (AT1 and AT2) in yeast cells. Confirmation of ROF2 interaction with the protein encoded by AT4 failed.

### 2.2. Site-Directed Mutagenesis of Immunophilins

When screening for CYP57 using Y2H, none of the interactors were identified and ROC3 resulted in three clones corresponding to two *A. thaliana* genes which were not confirmed by cotransformation.

As an explanation, it was proposed that the active enzymatic PPIase function likely leads to a conformational change in the protein structure of its partner during the interaction, and perhaps causes interaction complex dissociation. This, in turn, would alter the ability of yeast to grow on the selective medium. Thus, attempts to inactivate the active sites of the immunophilins were performed. In this regard, the web-based tool i-Tasser (Iterative Threading ASSEmbly Refinement) [19] was used to search in silico for possible activity-determining residues in the immunophilin amino acid sequences. i-Tasser was ranked as the best web-based server for protein structure prediction and utilizes state-of-the-art algorithms to identify structural templates from the protein databases by the multiple threading approach LOMETS (Local Meta-Threading Server). i-Tasser comparative results yielded evidence that His62 and Phe68 in ROC3 and His56 and Phe61 residues in CYP57 likely function as activity-essential positions. All aforementioned amino acids were replaced with Ala by in vitro mutagenesis. Two obtained mutant immunophilin forms were assigned as ROC3M and CYP57M. Due to the lack of well-established activity assays for these particular immunophilins, their actual functionality change was not validated at the current stage of the study. Similar active sites for ROF2 failed to be identified. However, this immunophilin was able to interact with the selected proteins in its native form (Table 1).

### 2.3. Interaction Partners for ROC3M

Y2H screening for ROC3M target proteins resulted in 112 clones that were selected according to ability to grow on QDO medium for ROC3M. After sequencing and the elimination of clones containing short (less than 200 bp) or shifted-ORF fragments, two candidates were selected. The results are shown in Table 2.

Using the same guidelines as for nonmodified PPIases, structural and ribosomal proteins were discarded, as well as the proteins showing nonspecific binding to multiple proteins (Table 2). Two selected proteins, At1g30580 and At1g12050, were cotransformed together with ROC3M into AH109 yeast cells to confirm their physical interaction.

**Figure 2:**
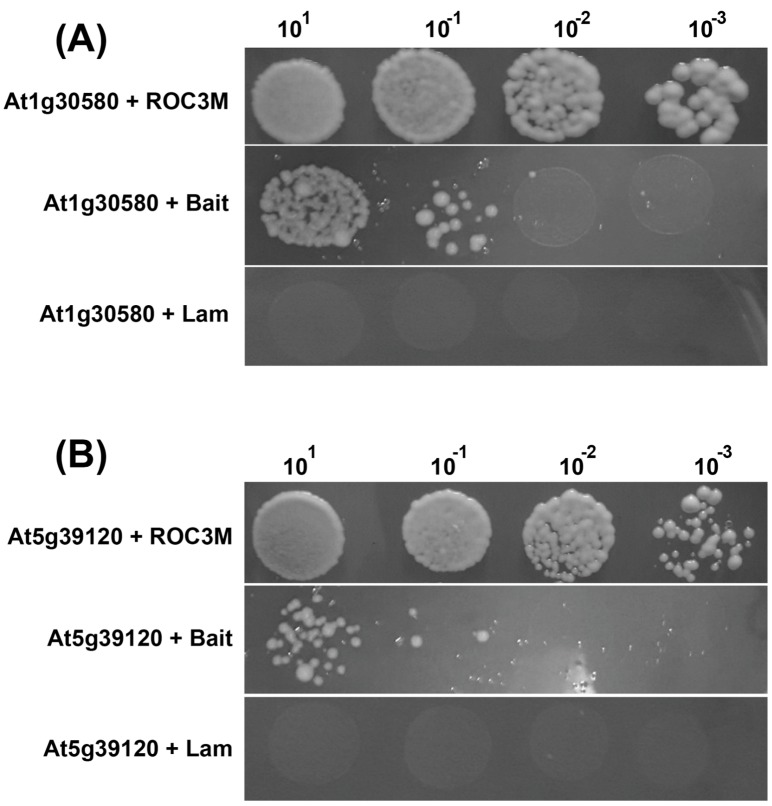
AH109 yeast strain cotransformation of ROC3M with At1g30580 (**A**) and At5g39120 (**B**), and two negative controls: the empty “bait” of empty pGBKT7 vector and pGBKT7 carrying the control gene of the human protein lamin C. The initial culture of OD600 = 0.2 was used, sequentially diluted to 10^−3^.

The results are shown in Figure 2A,B.

### 2.4. CYP57M Protein Partners

As a result of Y2H screening, 67 clones capable of growing on a QDO medium were selected. After sequencing and removal of samples containing short or shifted-ORF fragments, the following partner proteins for CYP57M were selected. The results are shown in Table 3.

**Figure 3:**
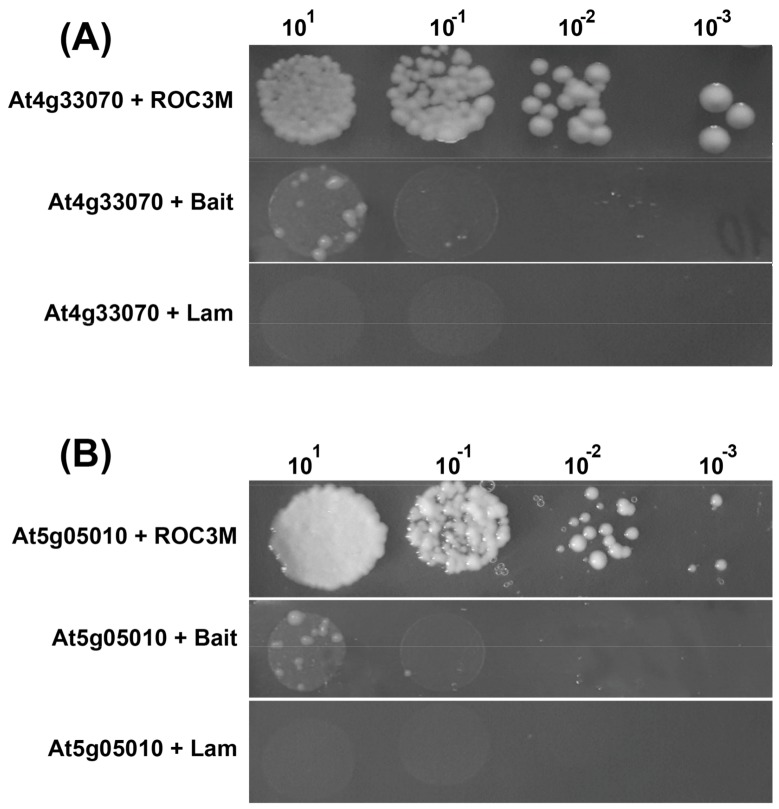
Cotransformation of CYP57M with At4g33070 (**A**) and At5g05010 (**B**) by the yeast two-hybrid system, and two negative controls: an empty bait vector pGBKT7 and pGBKT7 carrying the noninteracting human protein gene lamin C with CYP57M. The initial culture of OD600 = 0.2 was used, sequentially diluted to 10^−3^.

To test physical interaction with the modified PPIase CYP57M, proteins encoded by *At5g05010* and *At4g33070* were selected. The results are shown in Figure 3A,B.

## 3. Discussion

The first ROF2 interaction partner is AT1 (At1g70790; also known as C2-domain ABA-related 9 or CAR9), encoding the *A. thaliana* calcium-dependent lipid-binding domain-containing protein. According to the literature, AT1 interacts with the membrane receptor of abscisic acid (ABA) and regulates sensitivity to abscisic acid in *Arabidopsis* plants. This protein stimulates the activity of GTPase/ATPase Obg-like ATPases and provides intermediate calcium-dependent interaction of the abscisic acid receptors PYR/PYL/RCAR with the plasma membrane, and thus regulates the sensitivity to abscisic acid [20]. Furthermore, AT1 interacts with the ABA receptor PYR1-like 4, which is required for ABA-mediated responses, such as the closing of stomata and inhibition of seed germination via regulation of protein phosphatase group A (type 2C (PP2Cs)) activity [21,22]. ROF2 physical binding to AT1 can affect its function and/or its ability to interact with ABA-related partner proteins, and therefore, alter stress-response mechanisms.

As is known, the expression of large groups of genes is regulated by phytohormones. The main biotic stress-related plant hormone is salicylic acid (SA), whereas ABA regulates abiotic stress defense mechanisms. Traditionally, at the level of phytohormonal regulation, ABA functions as a complex together with antagonists SA, jasmonic acid (JA), and ethylene. Recent evidence suggests that ABA acts both as an activator and the suppressor of these protective ways [23,24,25]. The function of ABA is dependent on the time of infection and the nature of the pathogen [26]. In the early stages of defense against microbial invasion, ABA acts through the SA signaling pathway, stimulating stomatal closure and thereby reducing the possibility of infection [27]. After the penetration of pathogens, ABA is essential for callose accumulation induction, which has been shown to function as protection from fungal pathogens [26], while bacterial infection can block ABA-mediated production of callose [28].

Further evidence indicating the participation of АТ1 in callose deposition is its interaction with CALS5 (callose synthase 5, At2g13680.1), which is required for the formation of a callose wall separating tetraspores (interstitial wall) and surrounding the pollen mother cells, and was shown to be directly involved in the synthesis of callose during the growth of pollen [29].

It is also known that a violation of the ABA signaling pathway may improve the protection of plants against pathogens. One of the first works on molecular mechanisms of increased susceptibility to the fungal pathogen *Fusarium oxysporum* controlled by ABA was published by Anderson et al. [30].

As established earlier [1], ROF2 is localized predominantly in the nucleus of plant cells; although, according to the domain organization, it must also possess cytoplasmic localization. In addition, *Arabidopsis* transgenic plants with *ROF2* overexpression showed an increased accumulation of callose. This allowed the proposal of the relocation of this protein into the nucleus as a part of the protein complex, thus preventing AT1 interaction with membrane-bound ABA receptors modulating the response to pathogen invasion.

The second ROF2 interaction partner is At2g30020 (AT2; AP2C1), a putative protein phosphatase 2C 25, that negatively regulates defense responses via inactivation of MPK4 and MPK6 MAP kinases involved in stress signaling [31].

The ANPs–MPK6–MPK4 protein module is involved in the regulation of plant cytokinesis during meiosis and mitosis. In addition, МРK4 kinase controls the organization and stabilization of cortical microtubules as well as the development of root hair, and also negatively regulates SAR and SA-dependent defense signaling pathways. МРK6 kinase is involved in the oxidative stress signaling as well as in the innate immune MAR kinase-mediated response cascade (MEKK1, MKK4/MKK5, and MPK3/MPK6) launched by the flagellum bacterial receptor FLS2 [32,33]. МРK6 phosphorylates WRKY group transcription factors, and presumably is involved in hypersensitive response development control by phosphorylation of LIP5. Interestingly, transgenic *Arabidopsis* plants expressing *ROF2* (FKBP65) were characterized by increased expression of the WRKY33 gene [1]. Therefore, there is a possibility that AT2 phosphatase can act as a MAPK phosphatase, negatively regulating МАРK4 and МАРK6 kinases. This fact allows the proposal that ROF2 participates in the MAPK cascade, but is a part of the SA-independent pathway.

The ROC3M protein partner ENGD1 (encoded by the At1g30580 gene) belongs to the family of GTPase/GTP-binding proteins. Proteins of this family in plants negatively regulate the response to bacterial infection [34]. Overexpression of *OsYchF1* GTPase in *A. thaliana* plants resulted in a reduction of antioxidant enzymatic activity and increased lipid peroxidation, accompanied by accumulation of ROS. Representatives of this protein class in mammals are also involved in the control of stress responses. In particular, OBL1 (Obg-like ATPase) negatively regulates the response to oxidative stress [35]. The OLA1 gene inactivation leads to significant positive effects in protecting cells from peroxidation. In addition, OLA1 is not a part of signaling cascades and other known antioxidant mechanisms.

This evidence provided the opportunity to suggest that OLA1 may be an appropriate target to improve the protection of cells from peroxidation [35]. Together with earlier data [1], ROC3–ENGD1 interaction could alter the hydrogen peroxide level, resulting in a stronger defensive response of the plant. At5g39120, a RmlC-like cupin superfamily protein, represents another protein partner for ROC3M. RmlC (dTDP (deoxythymidine diphosphate)-4-dehydrorhamnose 3,5-epimerase) is a dTDP-sugar isomerase enzyme involved in the synthesis of l-rhamnose, a saccharide required for the virulence of some pathogenic bacteria [36]. RmlC is a dimer, each monomer being formed from two beta-sheets arranged in a beta-sandwich, where the substrate-binding site is located between the two sheets of both monomers. In Gram-negative bacteria, l-rhamnose is an important residue in the *O*-antigen of lipopolysaccharides, which are essential for resistance to serum killing and for colonization [36]. In Gram-positive bacteria, for example, l-rhamnose is known to be present in the capsule of *Streptococcus*, a causative agent of meningitis in humans. In *Streptococcus* mutants, l-rhamnose-containing polysaccharides have been implicated in tooth surface colonization and adherence to kidney, muscle, and heart tissues. In mycobacteria, l-rhamnose is fundamental to the structural integrity of the cell wall since it connects the inner peptidoglycan layer to the arabinogalactan polysaccharides.

Proteins of the RmlC-like family in rice are involved in antioxidant defense and detoxification during Cu-induced oxidative stress [37]. To overcome oxidative stress, plants often recruit enzymatic components such as superoxide dismutase (SOD), catalase (CAT), peroxidase (POD), and so forth. Protective responses against abiotic stress often go through the formation of active oxygen forms, and therefore, the ROC3 interaction with At5g39120 can regulate ROS accumulation in plant tissues to generate defensive response.

Next, CYP57M interacts with a protein encoded by *At4g33070*: pyruvate decarboxylase 1 (PDC1). This protein is involved in plant protective reactions during hypoxia. Under hypoxia, produced pyruvates are converted to acetaldehyde by PDC1, followed by reduction to ethanol and concomitant oxidation of NADH to NAD^+^. Thus, PDC1 plays a key role in alcohol fermentation. Overexpression of this gene in *Arabidopsis* plants improved the resistance of roots to hypoxia. Interestingly, transgenic plants constitutively expressing PDCI were shown to increase levels of soluble sugars, in conjunction with increased callose deposition and expression of PR genes resulting in impaired *Pseudomonas* infection [38]. This evidence of CYP57 involvement in the plant immune response to pathogen attack fully correspond to previous data on CYP57 [1].

The second interaction partner of CYP57M is a protein product of At5g05010: a coatomer subunit delta. The coatomer is a cytosolic protein complex that binds to dilysine motifs and irreversibly associates with Golgi non-clathrin-coated vesicles, which further mediate biosynthetic protein transport from the ER via the Golgi up to the trans-Golgi network. The coatomer complex is required for budding from Golgi membranes, and is essential for the retrograde Golgi-to-ER transport of dilysine-tagged proteins [39]. As is known, the sorting and transmembrane transport of proteins are important cellular mechanisms, especially under stress responses [40]. Indeed, coatomer proteins are involved in the control of PCD and maintenance of the ER [41,42]. Here, it is speculated that the CYP57 immunophilin can actively participate in these processes via interaction with PDC1.

## 4. Materials and Methods

### 4.1. Amplification and Cloning of Immunophilin cDNA Sequences and Identification of Interaction Partners

In the first phase of the study, to identify molecular partners with which ROC3, ROF2, and AtCYP57 interact in the plant cell, we utilized a yeast two-hybrid analysis (Y2H) for each target PPIase. Three “bait” vectors—plasmids pGBKT7 carrying the *ROC3*, *ROF2*, or *AtCYP57* gene—were mobilized into the yeast strain Y187. A total cDNA library of *A. thaliana* was cloned into the “prey” vector of pGADT7 and transferred to the yeast strain AH109. For this purpose, RNA was extracted from plants pretreated with flagellin (flg22), an integral modulator of biotic stress consisting of a 22-amino acid sequence and derived from the N-terminus of *Pseudomonas aeruginosa* flagellin, which causes the activation of the innate immune response in plants and animals specific to bacterial infection.

The following primers pairs were used to amplify:

- ROF2 sequence (5′–3′): FKB-For (*Eco*RI) CATACgaattcGAAGACGATTTCGACACGCA and FKB-Rev (*Sal*I) CATACgtcgacTCATGCCTTGGTGTCAATAC;
- ROC3 cDNA (5′–3′): ROC3-For (*Nde*I) CATACcatatgGCAACAAACCCTAAAGTCTA and ROC3-Rev (*Bam*HI) CATACggatccgAACCTCCACCTGTACATGTG;
- AtCYP57 (5′–3′): CYP-For (*Eco*RI) CATACgaattcTCGACGGTGTACGTGCTAGA and CYP-Rev (*Pst*I) CATACctgcagTCAGGCAAGAGATTTTCCAG.

As a template for PCR, the total cDNA obtained from the reverse transcription of the total *A. thaliana* RNA was used. Total RNA was extracted using an SV Total RNA Isolation System (Promega, Madison, WI, USA) and first-strand cDNA synthesis was done using the SuperScript III First-Strand Synthesis System (Invitrogen, Carlsbad, CA, USA), following the manufacturer’s recommendations. The indicated restriction sites were used for the cloning of PCR fragments into the bait vector pGBKT7 treated with corresponding nucleases. Sequencing of the obtained recombinant vectors was performed in order to verify the absence of ORF-shift mutations. Next, plasmids were transferred into *Saccharomyces cerevisiae* strain Y187 cells using an electroporation procedure according to the protocol for Matchmaker^®^ Gold Yeast Two-Hybrid System (Clonetech, Mountain View, CA, USA).

Prey vector pGADT7 containing an *A. thaliana* cDNA library was created using Matchmaker^®^ Gold Yeast Two-Hybrid System (Clonetech, Mountain View, CA, USA). To enrich the library of gene transcripts involved in defense responses, RNA was extracted from plant cells subjected to biotic stress: 3-h treatment of the 5-day-old *A. thaliana* seedlings and protoplast suspension with flagellin-22, and also of plants infected with *Pseudomonas syringae* DC3000 (3 days post-infection). Protoplasts were isolated from two hundred 10-day-old *A. thaliana* seedlings, harvested and incubated in 20 mL of solution containing 0.25% (*w*/*v*) macerozyme, 1.0% cellulase, 0.4 mm mannitol, 8 mm CaCl_2_, 5 mm MES-KOH (pH 5.6), and 0.1% bovine serum albumin for 14 h in the dark with gentle agitation at 40 rpm. Afterward, suspended protoplasts were filtered through a 100-μm cell filter, placed onto 20 mL of 21% (*w*/*v*) sucrose solution, and centrifuged at 300× *g* for 5 min resulting in clear fraction of protoplasts in the supernatant.

For infection, cultures of *P. syringae* DC3000 were grown overnight in LB medium at 25 °C up to OD600 = 1. Cells were centrifuged at 4500× *g* for 10 min and resuspended in infiltration buffer: 10 mM MES, 10 mM MgCl_2_, pH 5.8 at a concentration of 10^4^ cells/mL. The resuspended cells were pressure-infiltrated into the abaxial side of leaves.

Y2H analysis including all material preparation and growth conditions was conducted in accordance with the manufacturer’s recommendations for the Matchmaker Gold Yeast Two-Hybrid System (Clonetech, Mountain View, CA, USA).

### 4.2. Site-Directed Mutagenesis

Coding sequences of each ROC3 and AtCYP57 were amplified using two pairs of corresponding primers. One primer from each pair contained a substituted nucleotide resulting in amino acid transition to a neutral alanine in the final protein product: 

CYP-F (*Eco*RI) CATACgaattcTCGACGGTGTACGTGCTAGA and C57-iR AAGATGGTGTTGTCAAAGTAACC; and also C57m-F TTACTTTGACAACACCATCTTCGCTGCTGTCATTCCCGGTGCTCTCG and CYP-R (*Pst*I) CATACctgcagTCAGGCAAGAGATTTTCCAG; ROC3-F (*Nde*I) CATACcatatgGCAACAAACCCTAAAGTCTA and CY19-iR GCAAAGCTTGATCCTTTGTAGTG, and also CY19m-F ACTACAAAGGATCAAGCTTTGCCGCAGTGATTCCGAAAGCCATGTG and ROC3-R (*Bam*HI) CATACggatccgAACCTCCACCTGTACATGTG.

The obtained two PCR fragments for each gene were self-annealed by slow chilling from 95 °C to 50 °C with the rate of 1 °C per minute, followed by complete fragment extension with the use of Encyclo DNA polymerase (Evrogen.com) at 72 °C for 10 min in the presence of PCR buffer and the manufacturer-required concentration of dNTPs. Obtained DNA fragments were subjected to a second regular PCR round using one pair of terminal primers: CYP-F (*Eco*RI) and CYP-R (*Pst*I); ROC3-F (*Nde*I) and ROC3-R (*Bam*HI) in the following reaction conditions: denaturation at 95 °C for 10 s, annealing at 56 °C for 30 s, and extension at 72 °C for 60 s.

Incorporated restriction enzyme recognition sites were used to clone these PCR fragments into the bait vector pGBKT7 treated with the same corresponding enzymes, followed by transformation of recombinant plasmids into the Y187 yeast strain in accordance with the manufacturer’s recommendations for the Matchmaker Gold Yeast Two-Hybrid System (Clonetech, Mountain View, CA, USA).

### 4.3. Cotransformation of Yeast Cells

According to the Y2H screening data, full-length sequences of selected interactors were amplified for interaction verification with the use of the following primers presented in Table 4.

Restriction enzyme sites indicated in the primer sequences were used for PCR fragment treatment and cloning into the pGADT7 vector with compatible pretreated ends. Obtained recombinant pGADT7 vectors were mixed with bait plasmid pGBKT7 harboring one of the immunophilins and cotransformed simultaneously into the AH109 yeast strain, followed by the selection of transformants on DDO (SD/Leu/Trp) medium and interaction validation on QDO (Ade/His/Leu/Trp) medium. For both transformation and selection, protocols presented in the Matchmaker Gold Yeast Two-Hybrid System manual (Clonetech, Mountain View, CA, USA) were used. Bait vector pGADT7 expressing the negative control human lamin С protein was also provided by the manufacturer.

## 5. Conclusions

For all three immunophilins studied, protein partners were determined. The found molecular partners of PPIases were largely selected not chaotically, but by either direct relation to biotic and/or abiotic stress processes, or their direct or indirect interaction with known proteins and/or components of the plant defense mechanisms. At the same time, the results of previous work on the study of transgenic plants expressing immunophilins provided insight for this work, starting with the selection of candidate proteins and ending with the interpretation of results for the current obtained data in terms of PPIase partner protein participation in the control of plant immunity.

It is known that the protein expression and expression level depend on the stage of the response. Therefore, great benefits would be found by carrying out future research that refines and extends this work; namely, to make cDNA libraries at certain intervals after induction by flagellin, in order to identify PPIase partner proteins which are more specific to certain phases of the developing defense response.

## Figures and Tables

**Table 1:** Selected partner proteins for ROF2.

Number of Clones	Partner Protein	Cotransformation ID	Confirmation
2	Calcium-dependent lipid-binding domain-containing protein	At1g70790 (АТ1)	+
2	Putative protein phosphatase	At2g30020 (АТ2)	+
3	ATPase, F1 complex, OSCP/delta subunit	At4g00895 (АТ4)	−
1	60S ribosomal protein L27a-3 mRNA	False positive	N/A
1	40S ribosomal protein S20	False positive	N/A
1	H2A_12	False positive	N/A

**Table 2:** ROC3M interaction partners.

Number of Clones	Partner Protein	Cotransformation ID	Confirmation
2	ENGD-1, GTP-binding protein	At1g30580	+
1	RmlC-like cupin superfamily	At5g39120	+
1	At1g72370 40S ribosomal protein Sa-1	False positive	N/A
1	At2g36830 GAMMA-TIP, TIP1	False positive	N/A
1	At1g12050 putative fumarylacetoacetase	False positive	N/A
1	H2A_12	False positive	N/A

**Table 3:** Partner proteins of CYP57M selected in the process of Y2H screening. “+” shows true interaction.

Number of Clones	Partner Protein	Cotransformation ID	Confirmation
1	Putative pyruvate decarboxylase-1 Pdc1	At4g33070	+
1	Сlathrin adaptor complex medium subunit family protein At5g05010	At5g05010	+
1	At4g24280 chloroplast heat shock protein 70-1	False positive	N/A
1	At1g70800 ENHANCED BENDING 1	False positive	N/A
1	At3g53430 60S ribosomal Protein L12	False positive	N/A
1	At4g03520 thioredoxin M2	False positive	N/A
1	At1g70850 MLP-like protein 34	False positive	N/A
1	At2g36830 GAMMA-TIP, TIP1	False positive	N/A

**Table 4:** Partner proteins of CYP57M selected in the process of Y2H screening.

Gene ID	Forward Primer, 5′–3′	Reverse Primer, 5′–3′
ТОР1/At5g	TOP-For (*Nde*I) NNcatatgTTAATGGCGACTCCAACG	TOP-Rev (*Bam*HI) NNggatccTTAAGCAGAAGCAGAGGCAGC
АТ1/At1g70790	AT1-For (*Nde*I) NNcatatgGAAGATAAACCATTAGGGAT	AT1-Rev (*Xho*I) NNctcgagTTAGTCCAATCGTTTTGTCGGCA
AT2/At2g300020	AT2-For (*Nde*I) NNcatatgTCTTGCTCCGTCGCCGTA	AT2-Rev (*Bam*HI) NNggatccCTATATGAACTGGCGTAAAGG
At4/At4g00895	AT4-For (*Nde*I) NNcatatgGATACTCTCTCAGCATCC	AT4-Rev (*Bam*HI) NNggatccTCATCAAGAAACCCAGACAAG
At1g30580	At1g30580-For(*Eco*RI) gaatccATGCCTCCGAAAGCCAAAG	At1g30580-Rev(*Xho*I) ctcgagTCATTTCTTCCCACCACCGGA
At4g33070	At4g33070-For(*Eco*RI) gaattcATGGACACCAAAATCGGATC	At4g33070-Rev(*Xho*I) ctcgagCTACTGAGGATTGGGAGGAC
At5g05010	At5g05010- For(*Eco*RI) gaattcATGGTTGTGCTTGCTGCTG	At5g05010-Rev(*Bam*HI) ggatccTCATATGACTTGATAGTTCTGG
At5g39120	At5g39120-For(*Eco*RI) gaattcATGAAGGTGTCCATGTCTC	At5g39120-Rev(*Xho*I) ctcgagTTAGTTTTTAAACTTGGCCTC

## References

[1] Pogorelko G.V., Mokryakova M.V., Fursova O.V., Abdeeva I.A. (2014). Characterization of three Arabidopsis thaliana immunophilin genes involved in the plant defense response against Pseudomonas syringae. Gene.

[2] Laloi C., Appel K., Danon A. (2004). Reactive oxygen signalling: The latest news. Curr. Opin. Plant Biol..

[3] Fraire-Velázquez S., Rodríguez-Guerra R., Sánchez-Calderón L., Shanker A., Venkateswarlu B. (2011). Abiotic and Biotic Stress Response Crosstalk-Plants. Abiotic and Biotic Stress Response Crosstalk in Plants-Physiological, Biochemical and Genetic Perspectives.

[4] Spoel S.H., Dong X. (2008). Making sense of hormone crosstalk during plant immune response. Cell Host Microbe.

[5] Yasuda M., Ishikawa A., Jikumaru Y., Seki M., Umezawa T., Asami T., Maruyama-Nakashita A., Kudo T., Shinozaki K., Yoshida S. (2008). Antagonistic interaction between systemic acquired resistance and the abscisic acid-mediated abiotic stress response in Arabidopsis. Plant Cell.

[6] Bartoli C.G., Casalongué C.A., Simontacchi M., Marquez-Garcia B., Foyer C.H. (2013). Interactions between hormone and redox signaling pathways in the control of growth and cross-tolerance to stress. Environ. Exp. Bot..

[7] Nurnberger T., Brunner F., Kemmerling B., Piater L. (2004). Innate immunity in plants and animals: Striking similarities and obvious differences. Immunol. Rev..

[8] Bigeard J., Colcombet J., Hirt H. (2015). Signaling mechanisms in pattern-triggered Immunity (PTI). Mol. Plant..

[9] Cao X.Q., Jiang Z.H., Yi Y.Y., Yang Y., Ke L.P., Pei Z.M., Zhu S. (2017). Biotic and Abiotic Stresses Activate Different Ca^2+^ Permeable Channels in Arabidopsis. Front. Plant Sci..

[10] Jiang Z., Zhu S., Ye R., Xue Y., Chen A., An L., Pei Z.M. (2013). Relationship between NaCl- and H_2_O_2_-induced cytosolic Ca^2+^ increases in response to stress in rabidopsis. PLoS ONE.

[11] Espinosa-Diez C., Miguel V., Mennerich D., Kietzmann T., Sánchez-Pérez P., Cadenas S., Lamas S. (2015). Antioxidant responses and cellular adjustments to oxidative stress. Redox Biol..

[12] Schieber M., Chandel N.S. (2014). ROS function in redox signaling and oxidative stress. Curr. Biol..

[13] Torres M.A. (2010). ROS in biotic interactions. Physiol. Plant.

[14] Finkel T. (2011). Signal transduction by reactive oxygen species. J. Cell Biol..

[15] Rhee S.G. (2006). Cell signaling. H_2_O_2_, a necessary evil for cell signaling. Science.

[16] Asai S., Ohta K., Yoshioka H. (2008). MAPK signaling regulates nitric oxide and NADPH oxidase-dependent oxidative bursts in Nicotiana benthamiana. Plant Cell.

[17] Aumüller T., Jahreis G., Fischer G., Schiene-Fischer C. (2010). Role of prolyl cis/trans isomers in cyclophilin-assisted Pseudomonas syringae AvrRpt2 protease activation. Biochemistry.

[18] Singh K., Tzelepis G., Zouhar M., Ryšánek P., Dixelius C. (2018). The immunophilin repertoire of lasmodiophora brassicae and functional analysis of PbCYP3 cyclophilin. Mol. Genet. Genom..

[19] Iterative Threading ASSEmbly Refinement. https://zhanglab.ccmb.med.umich.edu/I-TASSER/.

[20] Rodriguez L., Gonzalez-Guzman M., Diaz M., Rodrigues A., Izquierdo-Garcia A.C., Peirats-Llobet M., Fernandez M.A., Antoni R., Fernandez D., Marquez J.A. (2014). C2-domain abscisic acid-related proteins mediate the interaction of PYR/PYL/RCAR abscisic acid receptors with the plasma membrane and regulate abscisic acid sensitivity in Arabidopsis. Plant Cell.

[21] Zhang X., Jiang L., Wang G., Yu L., Zhang Q., Xin Q., Wu W., Gong Z., Chen Z. (2013). Structural insights into the abscisic acid stereospecificity by the ABA receptors PYR/PYL/RCAR. PLoS ONE.

[22] Hao Q., Yin P., Li W., Wang L., Yan C., Lin Z., Wu J.Z., Wang J., Yan S.F., Yan N. (2011). The molecular basis of ABA-independent inhibition of PP2Cs by a subclass of PYL proteins. Mol. Cell.

[23] Asselbergh B., Achuo A.E., Höfte M., Van Gijsegem F. (2008). Abscisic acid deficiency leads to rapid activation of tomato defence responses upon infection with Erwinia chrysanthemi. Mol. Plant Pathol..

[24] Atkinson N., Urwin P.E. (2012). The interaction of plant biotic and abiotic stresses: From genes to the field. J. Exp. Bot..

[25] Fujita M., Fujita Y., Noutoshi Y., Takahashi F., Narusaka Y., Yamaguchi-Shinozaki K., Shinozaki K. (2006). Crosstalk between abiotic and biotic stress responses: A current view from the points of convergence in the stress signaling networks. Curr. Opin. Plant Biol..

[26] Ton J., Ent V.D.S., Hulten V.M., Pozo M., Oosten V.V., Loon L.C., Mauch-Mani B., Turlings T.C.J., Pieterse C.M.J. (2009). Priming as a mechanism behind induced resistance against pathogens; insects and abiotic stress. IOBC/Wprs Bull..

[27] Melotto M., Underwood W., Koczan J., Nomura K., He S.Y. (2006). Plant stomata function in innate immunity against bacterial invasion. Cell.

[28] Luna E., Pastor V., Robert J., Flors V., Mauch-Mani B., Ton J. (2011). Callose deposition: A multifaceted plant defense response. Mol. Plant Microbe Interact..

[29] Gomez J.F., Talle B., Wilson Z.A. (2015). Anther and pollen development: A conserved developmental pathway. J. Integr. Plant Biol..

[30] Anderson J.P., Badruzsaufari E., Schenk P.M., Manners J.M., Desmond O.J., Ehlert C., Maclean D.J., Ebert P.R., Kazan K. (2004). Antagonistic interaction between abscisic acid and jasmonate-ethylene signaling pathways modulates defense gene expression and disease resistance in Arabidopsis. Plant Cell.

[31] Schweighofer A., Kazanaviciute V., Scheikl E., Teige M., Doczi R., Hirt H., Schwanninger M., Kant M., Schuurink R., Mauch F. (2007). The PP2C-Type Phosphatase AP2C1, Which Negatively Regulates MPK4 and MPK6, Modulates Innate Immunity, Jasmonic Acid, and Ethylene Levels in Arabidopsis. Plant Cell.

[32] Lee Y., Kim Y.J., Kim M.H., Kwak J.M. (2016). MAPK Cascades in Guard Cell Signal Transduction. Front. Plant Sci..

[33] Ahmad P., Rasool S., Gul A., Sheikh S.A., Akram N.A., Ashraf M., Kazi A.M., Gucel S. (2016). Jasmonates: Multifunctional Roles in Stress Tolerance. Front. Plant Sci..

[34] Cheung M.Y., Li M.W., Yung Y.L., Wen C.Q., Lam H.M. (2013). The unconventional P-loop NTPase OsYchF1 and its regulator OsGAP1 play opposite roles in salinity stress tolerance. Plant Cell Environ..

[35] Zhang J., Rubio V., Lieberman M.W., Shi Z.Z. (2009). OLA1, an Obg-like ATPase, suppresses antioxidant response via nontranscriptional mechanisms. Proc. Natl. Acad. Sci. USA-Biol. Sci..

[36] Giraud M.F., Leonard G.A., Field R.A., Berlind C., Naismith J.H. (2000). RmlC, the third enzyme of dTDP-L-rhamnose pathway, is a new class of epimerase. Nat. Struct. Biol..

[37] Chen L., Liao B., Qi H., Xie L.J., Huang L., Tan W.J., Zhai N., Yuan L.B., Zhou Y., Yu L.J. (2015). Autophagy contributes to regulation of the hypoxia response during submergence in Arabidopsis thaliana. Autophagy.

[38] Tadege M., Bucher M., Stahli W., Suter M., Dupuis I., Kuhlemeier C. (1998). Activation of plant defense responses and sugar efflux by expression of pyruvate decarboxylase in potato leaves. Plant J..

[39] Xiang L., Etxeberria E., Van den Ende W. (2013). Vacuolar protein sorting mechanisms in plants. FEBS J..

[40] Carter C., Pan S., Zouhar J., Avila E.L., Girke T., Raikhel N.V. (2004). The Vegetative Vacuole Proteome of Arabidopsis thaliana Reveals Predicted and Unexpected Proteins. Plant Cell.

[41] Kursteiner O., Dupuis I., Kuhlemeier C. (2003). The Pyruvate decarboxylase1 Gene of Arabidopsis Is Required during Anoxia but Not OtherEnvironmental Stresses. Plant Physiol..

[42] Chen C., Song Y., Zhuang K., Li L., Xia Y., Shen Z. (2015). Proteomic Analysis of Copper-Binding Proteins in Excess Copper-Stressed Roots of Two Rice (Oryza sativa L.) Varieties with Different Cu Tolerances. PLoS ONE.

